# Effects on Gambling Activity From Coronavirus Disease 2019—An Analysis of Revenue-Based Taxation of Online- and Land-Based Gambling Operators During the Pandemic

**DOI:** 10.3389/fpsyt.2020.611939

**Published:** 2020-12-17

**Authors:** Anders Håkansson

**Affiliations:** ^1^Department of Clinical Sciences Lund, Psychiatry, Faculty of Medicine, Lund University, Lund, Sweden; ^2^Malmö Addiction Center, Region Skåne, Malmö, Sweden

**Keywords:** gambling disorder, online gambling, behavioral addiction, COVID-19, sports betting, online casino, land-based casinos

## Abstract

**Background:** Concerns have been raised about increased gambling problems during the coronavirus disease (COVID-19) crisis, particularly in settings with high online gambling and risks of migration from land-based to riskier online-based gambling types. However, few non-self-reported data sources are hitherto available. The present study aimed to assess changes in the online- and land-based gambling markets in Sweden during the first months affected by the societal impact of COVID-19.

**Methods:** Data were derived from national authority data describing monthly taxations of all licensed Swedish gambling operators, whose monthly tax payments are directly based on gambling revenue. Subdivisions of the gambling market were followed monthly from before COVID-19 onset in Sweden (mainly February 2020) through June 2020, when the sports market was restarted after COVID-19 lockdown.

**Results:** Overall revenue-based taxations in the licensed gambling decreased markedly from February to March, but stabilized onto an overall modest decrease through June. Commercial online casino/betting, despite some decrease in March, was maintained on a relatively stable level through June. However, within this category, horse betting increased steeply during the pandemic but returned to prepandemic levels later during the period. The state-owned operator in betting/online casino decreased markedly throughout the pandemic. The remaining commercial operators, mainly in online casino and online betting, demonstrated no change during the pandemic and ended on a June level 14% above the February level. Throughout the pandemic, the smaller restaurant casinos decreased markedly, while major state-owned casinos also closed entirely. State-owned lotteries and electronic gambling machines decreased markedly but were rapidly normalized to prepandemic levels.

**Conclusions:** Commercial online gambling operators' revenues remained stable throughout the pandemic, despite the dramatic lockdown in sports. Thus, chance-based online games may have remained a strong actor in the gambling market despite the COVID-19 crisis, in line with previous self-report data. A sudden increase in horse betting during the sports lockdown and its decrease when sports reopened confirm the picture of possible COVID-19-related migration between gambling types, indicating a volatility with potential impact on gambling-related public health.

## Background

Emerging research has highlighted that the coronavirus disease (COVID-19) pandemic may cause or worsen mental health problems (1) and that this may include addictive behaviors and addiction-like online behaviors (2, 3). Among the latter, problem gambling has been mentioned as a potential consequence of the pandemic and the restrictions surrounding it (4, 5).

Mechanisms potentially increasing gambling behavior during COVID-19 may include effects from the financial crisis and unemployment caused by the pandemic, but also, home confinement and changes in employment and everyday habits may enhance people's time at home and increase the time spent online. Likewise, the nearly total lockdown in sports events in most parts of the world during the early phases of the pandemic changed the gambling market significantly, logically leading to decreased gambling on sports events otherwise popular in gamblers (4). In March 2020, several countries took action in order to prevent a transfer to potentially more addictive types of gambling. Policy makers have expressed fear of gamblers switching to other gambling types, and that such a transfer in gambling habits may push gamblers or subpopulations of gamblers toward more rapid, online-based gambling types (6).

A self-report survey study in Sweden demonstrated that only a relatively limited minority of the population reported an increased gambling behavior during the early phases of the pandemic, but also that this subgroup had markedly higher rates of problem gambling than those reporting decreased or unaffected gambling (7). Likewise, another recent survey study from the same setting displayed findings in line with this; past-month gambling during the spring of 2020 was markedly lower for some gambling types compared to a previous report from the same setting, whereas some other gambling types appeared to be more preserved despite the pandemic. Typically, the more land-based gambling types were more affected by COVID-19-related lockdown and restrictions, whereas online gambling types appeared to be less affected (8). While such findings rely on self-reported data from survey respondents, objective data on gambling activities are needed, in order to demonstrate possible changes and transfers of gambling habits within the gambling market and between different types and modalities of gambling. One study on measured gambling data from one (anonymous) gambling operator demonstrated a modest impact from COVID-19 on gambling behavior, such that migration from sports betting to online casino within that specific operator could not be demonstrated (9). In contrast, however, a different study conducted in Ontario, Canada, demonstrated that some migration was likely between gambling types, due to the pandemic (10). Thus, findings are hitherto diverse with respect to the pandemic's consequences on gambling behavior, and further data are needed, if possible from objective sources of non-self-report gambling data.

Thus, the present study used official, national authority data on revenue-based taxation from gambling operators, aiming to study measures of financial activity in the overall legal gambling market in Sweden and the activity of specific subsections of the market. In the study, it was hypothesized that activity in the gambling market would have decreased in the gambling types related to sports events and other land-based gambling and that an increase may be possible in online-based gambling involving other types of gambling. Also, the study aimed to study whether decreases in some gambling types may be fully or partly counteracted by increases in other gambling types.

## Methods

### Variables

Data were derived from the Swedish Gambling Authority and included figures describing the monthly taxation of each of the gambling operators licensed for operation in Sweden. Gambling taxation equals 18% of the net revenue of the gambling operator (11) and is paid on a monthly basis to the Swedish national taxation authority. The data included in the present analysis can be applied for by individuals, media, and other organizations from the gambling authority or from the taxation authority and are available under the act making a broad range of governmental and authority documents and correspondence publicly available upon request. Data include no information on individual gamblers. Taxation from private companies in Sweden can be corrected up to 6 years later, for example in order to correct errors in the total revenue reported, but monthly revenue, and thereby the taxation level calculated from it, is considered a reliable source of gambling activity in the market, such that it is used by the Swedish Gambling Authority for the reporting of national gambling data.

Data were derived from the Swedish Gambling Authority during August 2020, when full monthly taxation data were available for all months from January 2019 (onset of the current regulation and taxation practices) through June 2020. Data used here included the months from January 2020 through June 2020. Data represented the full monthly taxation for each of the months, in Swedish currency (Swedish krona, 1 krona corresponding to around 0.10 Euros). For the best comparison possible, data were also reported for the same months of 2019, although direct comparisons are likely difficult, as the current gambling license system was introduced in January 2019 (11), such that the first months of 2019 may have been affected by the recency of new regulations at that time.

### Setting

Gambling operators are obliged to be licensed in Sweden in order to operate physically within the borders of Sweden or in order to operate online with their seat in Sweden. The registration in Sweden is mandatory for the use of Swedish money transfer instruments to a gambling account and for broadcasting in Swedish media sources (11). This legislation was initiated on January 1, 2019, and before that date, a large number of operators in the Swedish market were overseas companies operating from other EU countries but advertising and offering gambling in Swedish media channels and attracting a substantial proportion of Swedish gamblers. Thus, even before the introduction of the present legislation, online casinos were all operating from abroad while formally prohibited within Sweden, but still represented the most common gambling type reported by treatment-seeking gambling disorder patients (12) and the most common gambling type seen in television advertisements (13). Legal gambling types in Sweden, according to the updated legislation, are online casinos, land-based and online betting on sports and horse races, charity-based and other land-based and online lotteries (commercial and carried out by a large number of operators), land-based casinos in four major state-owned casinos across Sweden, and land-based electronic gambling machines (all state-owned in a monopoly situation), as well as a more limited market involving private restaurant-based casino games under a stricter regulation with limited deposits. In addition, relatively limited gambling activities in funfairs and similar events are legal but regulated. In the present study, charity-based lotteries are not included in the data. One state-owned gambling operator (*AB Svenska Spel*) operates in three subdivisions: in the monopoly position involving the four major land-based casinos and land-based gambling machines, and in two commercialized subdivisions operating in sports betting and online casino, and in lotteries and other chance-based number games, respectively. Thus, this state-owned company acts in a monopoly situation in some of these sectors (major land-based casinos and electronic gambling machines) and as one of the competing operators in the market of online casinos and sports betting. In the latter market, one further operator is the major organization of the Swedish horse racing industry (ATG, *Aktiebolaget Trav & Galopp*), which offers sports betting and online casino as well, but which traditionally plays a predominating role in land-based and now also online-based horse race betting.

An overall impression from the Swedish gambling market is the large share of online-based gambling in its exposition in advertisements (13) and in problem gamblers (12, 14). Trends in recent years have described a decreasing number of people reporting any past-year gambling (15), but a growing subgroup who fulfills the criteria of problem gambling in public health surveys. In total, between 1 and 1.5% of the adult population are believed to be moderate-risk problem gamblers, and the percentage of women among problem gamblers has been reported to increase in recent years (16). Also, while a majority of treatment-seeking patients with gambling problems are men (12), among online gamblers, female gamblers even have shown to have a higher risk of having gambling problems (14).

COVID-19 started to affect the Swedish society substantially during the month of March 2020 after a first case was detected in late January and the second case reported in late February. A series of events in mid-March marked the most dramatic impact of the pandemic, with travel restrictions, work-at-home recommendations, severe effects on financial markets, and restrictions on public gatherings. In parallel with this, during March, several major soccer leagues and other sports events were canceled or postponed, such that at the end of March, only a very limited number of less-known soccer leagues in the world were still operating. Thus, to an increasing extent throughout the month of March, in Sweden, the impression of COVID-19 consequences on society is likely to have been at its peak, with April leaving early recommendations unaltered and with a peak daily number of COVID-19-related deaths reported on April 7. In May, some major soccer leagues restarted and the opening of further European leagues was announced and thereafter took place in June. In mid-June, the Swedish soccer league reopened (Table 1).

**Table 1:** Time schedule of major COVID-19-related events in society and sports affecting Sweden.

**Month, 2020**	**Date and event**
January	January 31st—first confirmed case reported in Sweden
February	February 26th—second confirmed case reported in Sweden
March	March 9th—Italian male soccer league canceled March 10th—restriction of public gatherings to a maximum of 500 people in Sweden March 11th—first confirmed COVID-19 death in Sweden March 12th—historic baisse in the Stockholm stock market (−11%) March 13th—French male soccer league suspended March 14th—Swedish government advice not to travel to other countries March 15th—decision to suspend remaining ice hockey season in Sweden March 16th—advice to work at home, advice for elderly 70+ to stay at home in Sweden March 17th—advice to all high schools and universities in Sweden to conduct their studies online March 17th—decision to postpone Euro soccer 2020 for men March 19th—decision to postpone Swedish top-two leagues of male and female soccer March 20th—Volvo cars stop all production in Sweden March 20th—new crisis intervention from government to businesses in Sweden and a support intervention for sports and culture in Sweden March 24th—decision to postpone the 2020 summer Olympics in Tokyo March 27th—public gatherings of more than 50 people prohibited in Sweden March 30th—Swedish business newspaper *Dagens Industri* reports that the only soccer leagues still playing are Belarus, Nicaragua, Burundi, Turkmenistan, and Myanmar.
April	April 1st—international media report unexpected attention to low-tier Swedish soccer leagues due to lockdown of more established sports in Sweden April 7th—top number (through September, 2020) of COVID-19 deaths in Sweden
May	May 6th—announcement that male Bundesliga starts on May 16th May 9th—Korean soccer league started May 16th—start of German Bundesliga May 29th—decision that the highest league in male soccer in Sweden can start June 14th, other soccer leagues later May 29th—decision that La Liga Spain will start on June 11th
June	June 11th—start of Spanish La Liga June 14th—first game in Swedish top league in male soccer June 17th—first game in Premier league

While online casinos (and similar chance-based games, such as online bingo and similar games) and online sports betting represent different kinds of gambling, companies involved in any of them typically have a license for both, making it possible to offer gambling services in both these commercialized products, such that their respective share of the market cannot be easily separated from national market statistics. Thus, in the present analyses, and due to the large involvement of online gambling in Sweden, the subdivisions of the gambling market are reported as follows: state-owned land-based casino (one operator, state monopoly), limited-deposit land-based restaurant casinos (29 private operators), state-owned lotteries and electronic gambling machines (one state-owned operator), and commercial operators in online casino and online/land-based sports/horse betting (77 operators, including one state-owned operator, one private and predominating horse betting operator including land-based horse betting, and 75 private companies operating in online casino or online betting). The limited number of funfair games and similar events (three operators), lotteries and bingo operators without taxation data, and one private non-profit association organizing card games are not displayed in the table.

### Statistical Methods

Taxation data were compared, month by month, with respect to the full gambling taxation for the entire Swedish licensed gambling market and, also, with respect to the specific subtypes of gambling. In addition, data from minor gambling activities in festivals, funfairs, and similar events were reported for descriptive purposes. For each month of reported taxation, this was compared descriptively to the month of February, considered to be the last full month during which sports events, national financial markets, and public health were considered virtually unaffected by the COVID-19 pandemic (Table 2). As a comparison, the corresponding comparisons (each of the months, March through June, compared to the February levels) were also reported (Table 3). However, further statistical analyses were not carried out, as the present system of licensed gambling was introduced in January 2019. Thus, the corresponding period of time during 2019 also may not be fully representative, typically for gambling types which had previously been offered by overseas non-licensed companies and which were transferred to the legal gambling market in 2019.

**Table 2:** Revenue-based income taxes for Swedish licensed gambling operators, January–June, 2020 (approximated to thousands of Swedish krona, SEK).

	**January**	**February**	**March**	**April**	**May**	**June**
State-owned lottery/EGM (AB Svenska Spel, *n* = 1)	80,717	75,930	46,178	83,517	72,179	77,077
Commercial online casino + betting (*n* = 77)	237,123	219,734	201,266	209,702	223,921	216,213
- Principal horse betting operator AB Trav & Galopp (*n* = 1)	67,905	77,106	68,891	94,648	103,407	77,039
- State-owned Svenska Spel sports betting and online casino (*n* = 1)	40,744	37,877	20,862	12,138	17,708	19,908
- Other combined online casino/betting licenses (*n* = 75)	128,473	104,751	111,514	102,916	102,806	119,265
State-owned land-based casino (casino cosmopol, *n* = 1)	14,142	12,408	8,720	0	0	0
Land-based restaurant casino (*n* = 29)	3,397	3,501	2,510	1,888	2,074	1,910
Gambling for gifts (such as in funfairs and similar events, *n* = 3)	6,680	4,131	0	343	0	0
Total	335,388	311,583	258,681	295,113	298,180	295,199

*Data not displayed: bingo, land-based (one company, no taxation during the study period), charity lotteries (seven companies, no taxation during the study period), and one private non-profit organization with a land-based card game license (and with a taxation fluctuating slightly during the study period, with a 5% decrease from February to May but missing data for June)*.

**Table 3:** Revenue-based income taxes for Swedish licensed gambling operators, January–June, 2019 (approximated to thousands of Swedish krona, SEK).

	**January**	**February**	**March**	**April**	**May**	**June**
State-owned lottery/EGM (AB Svenska Spel, *n* = 1)	84,783	78,384	92,352	91,608	93,133	69,557
Commercial online casino + betting (*n* = 77)	195,748	202,408	213,792	216,443	209,594	201,090
- Principal horse betting operator AB Trav & Galopp (*n* = 1)	66,211	59,235	75,001	71,908	70,727	73,128
- State-owned Svenska Spel sports betting and online casino (*n* = 1)	33,904	35,438	34,494	29,853	30,273	23,037
- Other combined online casino/betting licenses (*n* = 75)	95,632	107,736	104,297	114,683	108,594	104,926
State-owned land-based casino (casino cosmopol, *n* = 1)	13,903	12,710	15,513	14,718	14,895	14,413
Land-based restaurant casino (*n* = 29)	2,353	2,677	3,524	3,330	2,996	3,101
Gambling for gifts (such as in funfairs and similar events, *n* = 3)	0	0	0	20,589	77,066	221,860
Total	296,787	296,179	325,180	326,119	320,700	288,383

*Data not displayed: bingo, land-based (one company, no taxation during the study period), charity lotteries (seven companies, no taxation during the study period), and one private non-profit organization with a land-based card game license (and with a taxation fluctuating slightly during the study period, with a 5% decrease from February to May but missing data for June)*.

## Results

### Overall Gambling Market

The overall level of gambling taxation decreased from February to March by 17%, but recovered partly in April to a level at 5% below the February level. At the end of the study period, in June, the overall level remained at 5% below the February level.

In the comparison year of 2019, the overall market increased in March, April, and May by 10, 10, and 8%, respectively, compared to the February level, whereas it decreased again in June 2019 to a level 3% below the February level of that year.

### Online Casino and Online/Land-Based Sports/Horse Betting

Commercial online casino/betting decreased by 8% from February to March, but within this category, the major horse betting operator decreased by 11% and the state-owned operator by 45%, whereas the remaining operators increased by 6%. Commercial online casino/betting thereafter increased in April to a level 5% below the February level; among these figures, the state-owned operator decreased further to a level 68% below the February level, whereas the major horse betting operator increased to a level 23% higher than the February level and the remaining operators decreased to a level 2% below the February levels.

In May and June, commercial online casino/betting stabilized on a level close to February levels (2% above in May and 2% below February levels in June). Through June, the state-owned operator recovered only partially, to a May level 53% below and a June level 47% below the February level. The major horse betting operator increased further in May, to a level 34% above the February level, but then decreased steeply in June, to nearly exactly the same level as in February. The remaining commercial online casino/betting operators remained in May on the same level as in April, i.e., 2% below the February level, but then increased in June, to a level 14% above the February levels.

In 2019, the overall market of commercial online casino/betting instead increased in March, April, and May, with 6, 7, and 4%, respectively, compared to the February level of that year, whereas it stabilized on a 1% decrease in June, compared to the February level. The major horse betting operator, in 2019, increased from February levels to 27, 21, 19, and 23%, respectively, in March through June. The state-owned operator, in 2019, saw a decrease compared to the February level of 3, 16, 15, and 35%, respectively, in March through June. Other remaining online casino/betting operators, in 2019, displayed figures fluctuating relatively little around the February level (a decrease of 3% in March, an increase of 6% in April, an increase of 1% in May, and a decrease of 3% in June).

### Lottery and Land-Based Casino/Electronic Gaming Machine Gambling

State-owned lottery/electronic gaming machine (EGM) gambling decreased by 39% from February to March and was thereafter normalized back to a level 10% above the February level in April and further normalized in May and June, compared to February levels (5% lower in May and 2% higher in June than in February). In 2019, these gambling types displayed, compared to the month of February, an increase of 17–19% in March through May, but a decrease in June of 11% compared to the February level.

Land-based casino gambling decreased from February to March by 30%, whereas state-owned casinos decreased to zero due to their full closure on April 1st, which was maintained throughout the study period. In 2019, when this gambling activity was obviously opened throughout the period, the values increased with 22% from the month of February to the month of March (while the levels of April, May, and June were 16, 17, and 13% above the February levels).

From February to March, restaurant casino gambling decreased by 28%, and from March to April, restaurant casino decreased further to a level 46% below the February level, whereafter it stabilized on a low level in May (41% below the February levels) and June (45% below the February levels). For this type of gambling, the values in 2019 demonstrated increases during March through June with 22, 16, 17, and 13%, respectively, compared to the February level.

## Discussion

The present study, assessing revenue-based taxation of gambling operators and thereby indirectly reflecting changes in the gambling market during the COVID-19 pandemic, indicates some relatively pronounced changes in the gambling market in Sweden, primarily from February to March 2020, i.e., in the transition where the pandemic increased steeply in the country and where land-based establishments in the society were the most affected, including the nearly total lockdown of elite sports events. Overall, the gambling market decreased markedly during the first pandemic-affected month studied, but recovered to a large extent, whereas the changes onto different subsections of the market were very diverse.

Primarily, the state-owned operator with a large involvement in sports betting decreased its revenues steeply, with a slow recovery over the next months, whereas online-based horse race betting increased substantially. In addition, major land-based casinos were completely closed on April 1st, and the smaller market of limited-stake restaurant-based casinos also decreased substantially. In contrast, however, the larger overall market of combined online casino and sports betting operators showed only a modest decrease, despite the sports lockdown. When excluding the major horse race operator and the state-owned operator, this subsection of the market decreased only marginally or even demonstrated some increase from February to March, despite the dramatic impact of the pandemic on sports betting.

Thus, the overall gambling market decreased only marginally and recovered relatively soon during the study period. However, it is evident from the present data that subsections of the gambling market need to be assessed separately; despite the dramatic impact on sports betting, the large subsection with license for both commercial online casino gambling and commercial sports betting remained relatively unaffected. Thus, although the exact amount of gambling cannot be established, this overall category remained relatively unaffected despite the lockdown of sports. Thus, in light of the decrease in sports events, it could be argued that the Swedish online-based market of chance-based games appears to be relatively robust, as indicated also, for example, by its predominating role in Swedish televised gambling advertisements (13). Thus, it can be at least assumed that chance-based gambling, such as online casino gambling, maintained a strong position even during the early phases of COVID-19. This is in line with the previous self-reported survey data in two general population studies (7, 8), and online casino is known to play a predominating role in problem gambling in the present setting; a majority of treatment-seeking clinical patients (12) and helpline callers (17) report online gambling to be their problematic gambling type. Thus, it can be suspected that in a setting with a strong role of chance-based online gambling, even the physical lockdown during COVID-19 affected gambling to a limited extent.

Interestingly, the major operator in horse race betting demonstrated a large increase, but also close to a normalization, already in the month of June, the month when international (and Swedish) soccer reopened, allowing for relatively normalized sports betting opportunities. Thus, it can be suspected that horse race betting was a short-term replacement of sports betting in the most acute phases of the pandemic, as horse races remained active throughout this period (although without physical audience on site). The increase in horse race betting is in line with a recent study, where online survey data from online gamblers in May 2020 demonstrated that online horse betting was one of the gambling types less affected by the pandemic than other types, and possibly even more commonly reported during the pandemic than in previous research using the same methodology (8). In another general population survey from the same setting, carried out in late April and early May 2020, only a relatively limited minority reported increasing horse betting as a response to the limited sports betting opportunities. Also, sports betting, both land-based and online-based, represented the gambling types most clearly affected by the pandemic; the number of respondents reporting decreased gambling for sports betting was almost 10 times larger than the number reporting an increase. In the same study, it was shown that gambling types less affected by the pandemic, according to self-report data, were online horse betting, online lotteries, and online casino gambling. In particular, in that general population study and when excluding people reporting to be non-gamblers for that particular type, 6 and 14% reported having increased land-based or online horse betting during COVID-19, respectively (7). Thus, the increase seen in horse race betting in the present study may seem consistent with the self-reported data on a partial migration from other gambling types to an increased horse betting. Likewise, however, the steep decrease in horse betting shown in the month of June may represent a rapid movement back in the opposite direction, such that the onset of some types of particularly soccer leagues and other major sports events in June may have attracted some gamblers back.

Also, the present findings are consistent with those in an early Australian online survey carried out in early April 2020, where a significant minority of respondents reported increased online gambling, although for all subtypes of gambling, reporting a decrease was more common than reporting an increase. While some decrease in overseas sports betting was seen, descriptions of transitions between gambling types were somewhat mixed, and conclusions hard to be drawn (18). While some survey data have indicated some migration—although limited—from sports betting to other gambling (7, 10), one study demonstrated the opposite. Auer and coworkers reported from one specific (and anonymous) online gambling operator that sports betting at that particular operator decreased in March 2020 and that their online casino gambling also decreased, arguing that in this particular (but unknown) gambling operator, no migration from sports to online casino gambling could be seen (9).

The Swedish land-based casino market is limited, and therefore, conclusions are difficult to draw about migration from these particular gambling types to others. The state-owned casinos (in the three largest cities of the country and in one regional urban center in mid-Sweden) closed on April 1, but also represented a limited share of the gambling market before that, and the more limited so-called restaurant casinos are small in comparison to the other types of gambling in the country. However, the latter gambling modality decreased clearly during the pandemic. It remains to be seen whether this effect is related to characteristics of the gambling pattern as such, or whether it is more associated with a likely decrease in restaurant visits during COVID-19 regulations. Again, the relatively limited size of these gambling types confirm the picture of the present setting as being particularly prone to online gambling, and that crisis-related changes in the market are likely to occur within the large share of online-based gambling options.

Although the overall effect of COVID-19 on gambling behavior may be modest but largely negative, it cannot be excluded that when gambling is only modestly decreasing, such as in the present study, some individuals may increase their gambling and the health hazards occurring to them may be significant. In one of the online surveys carried out in the present setting, a minority reported that they increased online casino gambling in response to the decrease in sports betting, or that they increased other sports betting in contrast to sports seeing a decrease, and among them, rates of gambling problems were very large and even predominant (7). Thus, the public health effects from COVID-19-related changes in the gambling market may be highly diverse and could paradoxically deteriorate gambling behavior in some individuals and improve a problematic gambling behavior in others.

The sole period of time available for comparison to the present study period is the corresponding season during 2019, such that effects of seasonality (diverse sports events and climate- and weather-related differences) can be assessed only with that comparison. However, this comparison must be judged difficult, as 2019 was the very first year in a novel license system, where previously overseas operators and markets had become legal and licensed in Sweden as late as in January 2019. However, when comparing, the overall gambling taxation for the whole license marked did not demonstrate a decrease from February to March in 2019, making it likely that this relatively pronounced decrease in March 2020 can be attributed to the effects of COVID-19. However, in 2020, the market recovered partly and ended on a June level which differed from that year's February level in a manner comparable to the 2019 change from February to June.

However, the commercial sports betting and online casino division of the state-owned operator, AB Svenska Spel, demonstrated a decrease from February through June 2019 of around 35%. Thus, based on the latter, it cannot be excluded that some part of the decrease during the present study's study period may derive from changes also occurring in the same season in 2019, but again, data are difficult to compare, as 2019 was the very 1st year with the present system and, therefore, may not provide a representative picture. However, the predominating horse betting operator, which in the present study demonstrated some decrease in March and a marked increase in April and May, demonstrated a 27% increase from February to March in 2019, whereafter the same level remained stable through June. Thus, the large fluctuations in horse race betting during COVID-19 are unlikely to be explained by any factor also occurring in 2019 and, therefore, may be more likely to be related to actual changes in the pandemic. The remaining commercial betting/online casino operators altogether demonstrated relatively stable figures from February through June in 2019, with a 3% decrease in June compared to February. Thus, although again 2019 is the sole and possibly limited opportunity for actual comparison, the stable but slightly increasing trend in these operators from February to June in 2020 is comparable to 2019, or even indicating a potential increase. Again, this comparison also does not clearly reveal other explanatory factors to the development seen during COVID-19.

For land-based restaurant casinos, the operators available did not demonstrate a decrease in 2019 similar to the one seen during the COVID-19 period; instead, all months from March through June in 2019 saw higher values then in February in this category, such that changes seen in the present study also may not be attributable to season-related changes but may be related to COVID-19-related factors. Thus, also for this category, changes in the present study cannot readily be explained by historic factors such as seasonality.

The present study may have implications for preventive work and for harm reduction programs in gambling, as well as for future research. Self-exclusion is one of the harm reduction options for individuals with a problematic gambling behavior (19, 20), and there is growing evidence in therapeutic interventions in gambling disorder (21, 22). Thus, in case of a growing gambling problem due to the COVID-19 pandemic or other similar crises, actions from stakeholders can involve efforts to increase early detection, self-exclusion from gambling, and structured gambling disorder treatment. Thus, beyond the macrolevel effects on gambling markets, they demonstrate a certain likelihood that subpopulations may present worsening symptoms during this crisis, calling for earlier intervention in those changing their gambling habits in response to this specific crisis.

The current study has limitations and strengths. It uses a novel source of information describing the activity in the gambling market, i.e., the level of taxation based on the financial revenue of gambling operators, thereby theoretically reflecting the true level of gambling within the country, rather than data reported in previous self-report surveys (7, 8). Likewise, limitations include the fact that the data used do not reflect actual gambling activity from the perspective of individual gamblers, but instead from the perspective of the gambling operator; while the level of profit of the company may change from month to month because of hazard-based outcome actual gambling events, these differences are likely to be limited. Also, the possibilities of further statistical analyses were limited, due to the fact that regarding potential seasonality of gambling activity, data could be compared only to the corresponding months in the previous year, as the current system of legal, licensed gambling was introduced as late as in January 2019. Also, it could be suspected that the first months of such a system may not be fully representable. For these reasons, a statistical analysis, such as a formal time series analysis involving both 2020 and months from 2019 which were comparable to a limited extent, was not carried out. In addition, taxations can theoretically be corrected up to 6 years later, although this is likely to be rare and to have a very limited impact on the monthly trends in taxation.

## Conclusions

Based on revenue-based taxations of licensed gambling operators, in total and for subsections of the gambling market, the present study demonstrates that some substantial changes in gambling activity occurred during early COVID-19 in Sweden, although changes appeared to be normalized to a large extent already during the third full month of COVID-19-related restrictions in the country. In the present online-based gambling markets, commercial operators' revenues were little affected by the steep decrease in sports events, likely because of the parallel online casino involvement of these companies. Instead, theoretically decreased sports betting may instead transfer a substantial proportion of gambling to horse races while these were not canceled during the pandemic. The relative stability of the total gambling market activity in a highly online-based gambling market is noteworthy, given the nearly total cancelation of sports during the early COVID-19 phases. This calls for further research in order to understand possible transitions across gambling types in times of crisis.

## Data Availability Statement

Publicly available datasets were analyzed in this study. This data can be found here: Data are publicly available upon request to Swedish authorities, such that data of the present kind are typically presented as follow-up data by the Swedish Gambling Authority. Thus, the present data can be requested from that authority, or indirectly through the author.

## Author Contributions

The author confirms being the sole contributor of this work and has approved it for publication.

## Conflict of Interest

AH has an overall research support from the state-owned gambling operator Svenska Spel, and from the research council of that body and the research councils of the Swedish alcohol monopoly and the Enforcement Authority, as well as from the regional hospital organization. None of these organizations had any role in or influence on the present work.
